# Cancer survival differences between European populations: the UK uneasiness

**DOI:** 10.1054/bjoc.2001.1999

**Published:** 2001-09

**Authors:** G Gatta, R Capocaccia, F Berrino

**Affiliations:** Division of Epidemiology, Istituto Nazionale per lo Studio e la Cura dei Tumori, Milan, Italy; Laboratory of Epidemiology and Biostatistics, Istituto Superiore di Sanità, Rome, Italy


*British Journal of Cancer* (2001) **85**(6), 785–786
© 2001 Cancer Research Campaign
doi: 10.1054/ bjoc.2001.1999, available online at http://www.idealibrary.com on IDEAL

Editorial

### Cancer survival differences between European populations: the UK uneasiness

In no country has the impact of EUROCARE (Berrino et al, 1995, 1999; Coebergh et al, 1998; Capocaccia et al, 2001) – which provides a comparative analysis of cancer survival in Europe – been so great as the UK. This is doubtless due to the surprisingly low level of survival found for British patients compared to those in most other western European countries. And it is understandable that a debate on the interpretation and validity of the EUROCARE results should have arisen. The paper by Woodman and co-workers (2001) in this issue is a further contribution to this debate. It presents a detailed critique of some of the methods of EUROCARE and concludes that survival data from many European cancer registries may be flawed so it would be more appropriate to compare UK survival data (which is not flawed) with that from Scandinavian countries (which is also reliable). Nevertheless Woodman et al (2001) concede that UK survival is worse than that of most Scandinavian countries and argue for immediate measures to identify and rectify suboptimal care.

One issue raised indirectly by the paper of Woodman et al (2001) is whether international comparison of survival, based on cancer registry data, is useful at all, because different registries use different diagnostic procedures, different follow-up procedures and have variable completeness of coverage. In particular Woodman et al suggest that survival may be inflated in some registries because of failure to identify all cases of advanced disease. This is an important issue but one that has been fully addressed in EUROCARE publications. These ‘incompatibilities’ can be analysed individually and their impact on survival figures estimated. It is found that the confounders have a small effect on the data and intercountry differences in survival persist after taking account of them (Berrino et al, 1995, 1999, 2001). It is noteworthy that intercountry survival differences reduce or even disappear altogether for cancers such as testicular cancer, Hodgkin’s disease, and several childhood cancers, for which highly effective treatments exist. If the survival differences observed for the major cancer sites – colon, rectum, melanoma, breast, prostate – are due to registration or follow-up bias, one wonders why similar differences are not observed for all cancer sites.

The assertion of Woodman et al that UK survival rates can only reliably be compared with those of Finland and Scandinavia, reflects, in our opinion, a prejudice that the quality of incidence and survival data is lower in southern European countries. There is no evidence to support this prejudice. Many southern European cancer registries have quality and exhaustiveness indicators as good as the best in northern Europe (Parkin et al, 1997). It is true that cancer registration developed later in southern Europe, and small regional registries were set up rather than national ones; but, exploiting the experience of the longer-established registries, they set-up links with numerous sources of information from the outset, usually including population files of residents, and implemented efficient systems of active quality control and follow-up (Berrino et al, 1995, 1999; Parkin et al, 1997). Of course, the extent to which a series of small registries covering a small fraction of the national territory provides a representative picture of the whole country remains an issue – as repeatedly emphasised in EUROCARE publications. In Italy, for instance, cancer patients’ survival is higher in the north of the country, where coverage is also higher (Veradecchia et al, 1997). Ecological techniques to model national data from the data provided by local registries have recently been proposed (Mariotto et al, 2001). In any event the issue of national representativeness should not be allowed to cast doubt on the legitimacy of comparing incidence and survival between population-based registries.

A further criticism concerns the difficulty of interpreting survival differences because of missing information on stage and other clinical variables. The high resolution (HR) studies that collect standardised information on stage, staging procedures and treatment from small samples of incident cases were conceived to address this issue. HR studies permit stage-specific and stage-adjusted survival comparisons corrected for stage migration, i.e. corrected for the fact that staging examinations, practices and facilities vary. However, they are expensive and difficult to carry out because they require direct access to clinical records. Only a few registries have such access or the resources to gather information from them. In HR studies, the cases examined do not have to be representative of the national population, but should be representative of the registry population, as recognised in the Woodman paper. To ensure this, HR studies should be nested with general survival studies.

So far cancer registries have only been able to submit small numbers of cases for HR studies. As a consequence, the results are uncertain and the conclusions preliminary. The paper by Woodman et al draws attention to several inconsistencies between survival rates estimated from a few hundred cases included in the recent HR study (Gatta et al, 2000) and those obtained from many thousands of cases diagnosed in a previous period and included in the main EUROCARE II study. It is right that such inconsistencies should be pointed out; in fact they were not unexpected given that the study periods differ, and survival rates are changing. These authors highlighted in particular that in the HR study (Gatta et al, 2000) the population of the Modena registry did not perfectly coincide with that presented in the EUROCARE II monograph.*

Clearly further HR studies on larger samples are required to clarify how much intercountry survival differences are due to differential delay in diagnosis and how much to differences in treatment.

To return to more general matters. Some authors feel that monitoring other types of data is more useful than monitoring survival, or even that population-based survival data are useless (Irwig et al, 2000; Peto et al, 2000). Our opinion is that mortality data and adequacy of treatment data are useful, but cannot be fully interpreted without corresponding survival data. Moreover, mortality and treatment data are not without defects. It is true, however, that survival figures are often published after a long delay, and this is unfortunate when short-term survival is informative. EUROCARE is moving toward a more timely centralisation and analysis of survival data.

*In this paper (Gatta et al, 2000) the HR data for Modena were restricted to cases diagnosed in 1990–91 and from a smaller (urban) area than that considered in EUROCARE II (1985–1989) which included cases diagnosed in the whole province in 1988–89. The HR study was in fact confined to the Modena colorectal cancer registry which covers only part of the province. The incidence in 1990–91 was 315 cases, 306 of which were eligible for inclusion in the study (Ponz de Leon et al, 1998).

To conclude, Woodman et al acknowledge that cancer survival is poorer in the UK than in the countries they consider worthy comparators, and suggest that access to effective treatment is important, recommending systematic audits of treatment quality. We agree that audit studies are important, but in order to inform the interpretation of future mortality and survival trends, they should be population-based – and this will be even more difficult than carrying out retrospective registry-based studies of patterns of care, as done by EUROCARE. Finally, whatever action the UK government takes to improve cancer survival, population-based survival studies will be a necessary check that their intervention has been effective.

Woodman et al (2001) also correctly point out an inconsistency between the observed and relative survival figures. The Gatta et al paper (Gatta et al, 2000) erroneously reported the differences between observed and relative survival. In fact, differences at three years were larger than reported but the rank of countries did not change and the hazard ratios based on relative survival changed only slightly. We preferred to present the results with the more familiar Cox model also due to its higher statistical power.

**G Gatta[1], R Capocaccia[2] and F Berrino[1]**
**(e-mail: gatta@anprisc.anapat.istitutotumori.mi.it)**

[1]Division of Epidemiology, Istituto Nazionale per lo Studio e la Cura dei Tumori, Milan, Italy; [2]Laboratory of Epidemiology and Biostatistics, Istituto Superiore di Sanità, Rome, Italy

### REFERENCES

Berrino F, Sant M, Verdecchia A, Capocaccia R, Hakulinen T and Estève J (1995) *Survival of cancer patients in Europe. The EUROCARE Study*. IARC Scientific Publication No 132. Lyon: International Agency for Research on Cancer

Berrino F, Capocaccia R, Estève J, Gatta G, Hakulinen T, Micheli A, Sant M and Verdecchia A (1999) *Survival of cancer patients in Europe: the EUROCARE Study II*. IARC Scientific Publication No 151. Lyon: International Agency for Research on Cancer

Berrino F, Gatta G, Sant M and Capocaccia R (2001) The EUROCARE study of survival of cancer patients in Europe: aims, current status, strengths and weaknesses. *Eur J Cancer* **37**: 673–677

Coebergh JWW, Sant M, Berrino F and Verdecchia A (1998) Survival of adult cancer patients in Europe diagnosed from 1978–1989: the Eurocare II Study. *Eur J Cancer* **34**: 14

Capocaccia R, Gatta G, Magnani C, Stiller C and Coebergh JWW (2001) Childhood Cancer Survival in Europe 1978–1992: the Eurocare Study. *Eur J Cancer* **37**(6)

Gatta G, Capocaccia R, Sant M, Bell CMJ, Coebergh JWW, Damhuis R, Faivre J, Martinez Garcia C, Pawlega J, Ponz de Leon M, Pottier D, Raverdy N, Williams EMI and Berrino F (2000) Understanding variations in survival for colorectal cancer in Europe: a EUROCARE High-Resolution Study. *Gut* **47**(4): 533–538

Irwig L and Armstrong B (2000) EUROCARE-2: relevance for assessment of quality of cancer services? *Lancet* **355**: 427–428

Mariotto A, Capocaccia R, Verdecchia A, Micheli A, Feuer EJ, Pickle L and Klegg L (2001) Projecting SEER cancer survival rates to the US: an ecological approach. CCC, accepted for publication

Parkin DM, Muir CS, Whelan SL, Ferlay J, Raymond L and Young J (1997) (eds) Cancer Incidence in Five Continents, Vol VII. IARC Scient Publ No 120, Lyon: International Agency for Research on Cancer

Peto R, Boreham J, Clarke M, Davies C and Beral V (2000) UK and USA breast cancer death down 25% in year 2000 at ages 20–69 years. *Lancet* **355** (1822)

Ponz de Leon M, Di Gregorio C, Roncucci L, Benatti P, Fante R, Rossi G, Foroni M, Pedroni M and Sassatelli R (1998) Epidemiology of tumours of colon and rectum. Incidence, mortality, familiality and survival in the health care district of Modena 1984–1995. Università di Modena

Verdecchia A, Micheli A and Gatta G (1997) (eds) Survival of Cancer Patients in Italy. The ITACARE Study. *Tumori* **83**: No.1

Woodman CBJ, Gibbs A, Scott N, Haboubi NY and Collins S (2001) Are differences in stage at presentation a credible explanation for reported differences in the survival of patients with colorectal cancer in Europe?

*© 2001 Cancer Research Campaign*

